# Editorial: Exploring exopolysaccharides: probiotic pathways from gut to brain health

**DOI:** 10.3389/fmicb.2026.1826689

**Published:** 2026-04-17

**Authors:** Sergio D'Ambrosio, Susana Langa, Donatella Cimini

**Affiliations:** 1 Department of Environmental, Biological and Pharmaceutical Sciences and Technologies, University of Campania Luigi Vanvitelli, Caserta, Italy; 2 Food Technology Department, National Institute for Agricultural and Food Research and Technology (INIA-CSIC), Madrid, Spain

**Keywords:** exopolysaccharides (EPS), gut-brain axes, microbial metabolites, neuroplasticity, probiotic

The interaction between the gastrointestinal tract and the central nervous system, known as the gut-brain axis (GBA), has emerged as a transformative field in biomedical research (Cryan and Dinan, 2012). Probiotics—beneficial live microorganisms—and postbiotics—non-viable microbial products such as exopolysaccharides (EPS) and bacteriocins—are at the forefront of this revolution. Their ability to modulate neural function, immune responses, and metabolic pathways offers promising therapeutic avenues for disorders ranging from stress and depression to epilepsy and neurodegeneration. This Research Topic, “*Exploring Exopolysaccharides: Probiotic Pathways from Gut to Brain Health*,” compiles cutting-edge research that deepens our understanding of these mechanisms, bridging the gap between fundamental microbiology and clinical application.

To fully grasp the therapeutic potential of these interventions, it is essential to understand the underlying biological mechanisms. In their comprehensive review, Al Noman et al. explore the intricate relationship between the microbiome and neuroplasticity. They detail how microbial metabolites, including short-chain fatty acids (SCFAs) and tryptophan derivatives, alongside immune and hormonal regulators, influence brain reorganization. Their work highlights that dysbiosis is not merely a symptom but a potential driver of neurodevelopmental and psychiatric conditions, a concept strongly supported by the critical role of microbial metabolites in gut-brain communication (Dalile et al., 2019), suggesting that targeted microbiome interventions could enhance cognitive flexibility and mental health resilience.

Translating these mechanisms into clinical practice requires rigorous evidence, particularly for complex neurological disorders. Tan et al. provide a significant contribution with a meta-analysis evaluating the efficacy of probiotics as an adjunctive treatment for epilepsy. By synthesizing data from randomized controlled trials (RCTs), they demonstrate that combining probiotics with antiepileptic drugs significantly reduces seizure frequency and duration compared to standard therapy alone. Furthermore, their analysis reveals improvements in electroencephalogram (EEG) patterns and intestinal barrier function markers, reinforcing the hypothesis that gut health is intrinsically linked to neurological stability, an area of growing focus even in the management of refractory epilepsy (Lum and Olson, 2020).

Beyond neurological pathology, probiotics also hold promise for managing psychological wellbeing in the general population. In a randomized controlled trial focusing on healthy young adults, Noorwali et al. investigated the effects of *Lactobacillus rhamnosus* GG (LGG) on perceived stress. Their findings indicate that a 30-day supplementation course significantly reduced stress scores, particularly in male participants. This study underscores the potential of probiotics as a safe, accessible strategy for stress management, highlighting the sensitivity of the GBA to microbial modulation even in the absence of clinical disease.

While clinical outcomes are encouraging, the discovery of novel bioactive compounds remains the engine of innovation in this field. Ravi and Pan present compelling data on *Lactiplantibacillus pentosus* C82, a strain isolated from fermented cabbage. Their study characterizes the strain's ability to produce bacteriocins and exopolysaccharides (EPS) that effectively inhibit biofilm-forming pathogens like *Escherichia coli* and *Serratia marcescens*. Using a zebrafish model, they demonstrated that these bioactive metabolites not only mitigate gut dysbiosis but also reduce inflammation and restore antioxidant defenses. This research exemplifies how exploring non-conventional sources can yield potent postbiotics with dual benefits for gut integrity and systemic health, expanding on the well-documented health-promoting properties of bacterial exopolysaccharides (Caggianiello et al., 2016).

Collectively, the articles in this Research Topic illustrate the multi-dimensional impact of probiotics and their derivatives. From modulating neuroplasticity and managing stress to treating epilepsy and combating pathogenic biofilms, the findings presented here advance our knowledge of the gut-brain axis. As we look to the future, the integration of detailed molecular characterization of EPS with robust clinical trials will be key to unlocking the full therapeutic potential of the microbiome for human health.
